# {μ-6,6′-Dimeth­oxy-2,2′-[propane-1,3-diylbis(nitrilo­methyl­idyne)]diphenolato}dimethano­ltrinitratonickel(II)samarium(III) methanol disolvate

**DOI:** 10.1107/S1600536808038920

**Published:** 2009-01-08

**Authors:** Fei Liu

**Affiliations:** a The College of Chemical Engineering & Materials, Eastern Liaoning University, No. 325 Wenhua Road, Yuanbao District, Dandong City, Liaoning Province 118003, People’s Republic of China

## Abstract

In the title complex, [NiSm(C_19_H_20_N_2_O_4_)(NO_3_)_3_(CH_4_O)_2_]·2CH_3_OH, the Ni^II^ ion is coordinated by two O atoms and two N atoms of a deprotonated Schiff base ligand and by two O atoms of two methanol ligands in a slightly distorted octa­hedral geometry. The Sm^III^ ion is coordinated by six O atoms from three chelating nitrate ligands and four O atoms from a Schiff base ligand in a distorted bicapped square-anti­prismatic environment. In the crystal structure, inter­molecular O—H⋯O hydrogen bonds connect complex mol­ecules and methanol solvent mol­ecules, forming (10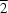) sheets.

## Related literature

For the isostructural Pr(III) complex, see: Liu & Zhang (2008 ▶). For a related Sm(III) complex, see: Wang *et al.* (2008 ▶). 
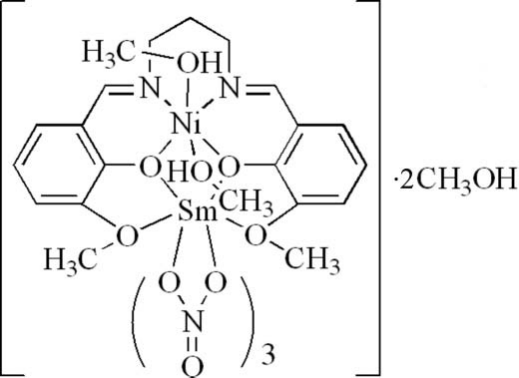


## Experimental

### 

#### Crystal data

[NiSm(C_19_H_20_N_2_O_4_)(NO_3_)_3_(CH_4_O)_2_]·2CH_4_O
*M* *_r_* = 863.63
Monoclinic, 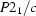

*a* = 13.066 (3) Å
*b* = 11.121 (2) Å
*c* = 22.128 (4) Å
β = 90.60 (3)°
*V* = 3215.2 (11) Å^3^
*Z* = 4
Mo *K*α radiation
μ = 2.48 mm^−1^
*T* = 291 (2) K
0.21 × 0.20 × 0.18 mm

#### Data collection

Rigaku R-AXIS RAPID diffractometer
Absorption correction: multi-scan (*ABSCOR*; Higashi, 1995 ▶) *T*_min_ = 0.619, *T*_max_ = 0.660
22609 measured reflections
7354 independent reflections
6200 reflections with *I* > 2σ(*I*)
*R*_int_ = 0.035

#### Refinement

*R*[*F* ^2^ > 2σ(*F* ^2^)] = 0.027
*wR*(*F* ^2^) = 0.063
*S* = 1.03
7354 reflections
430 parameters
18 restraints
H-atom parameters constrained
Δρ_max_ = 0.58 e Å^−3^
Δρ_min_ = −0.38 e Å^−3^

### 

Data collection: *RAPID-AUTO* (Rigaku, 1998 ▶); cell refinement: *RAPID-AUTO*; data reduction: *CrystalStructure* (Rigaku/MSC, 2002 ▶); program(s) used to solve structure: *SHELXS97* (Sheldrick, 2008 ▶); program(s) used to refine structure: *SHELXL97* (Sheldrick, 2008 ▶); molecular graphics: *SHELXTL* (Sheldrick, 2008 ▶); software used to prepare material for publication: *SHELXL97*.

## Supplementary Material

Crystal structure: contains datablocks global, I. DOI: 10.1107/S1600536808038920/lh2735sup1.cif

Structure factors: contains datablocks I. DOI: 10.1107/S1600536808038920/lh2735Isup2.hkl

Additional supplementary materials:  crystallographic information; 3D view; checkCIF report

## Figures and Tables

**Table 1:** Hydrogen-bond geometry (Å, °)

*D*—H⋯*A*	*D*—H	H⋯*A*	*D*⋯*A*	*D*—H⋯*A*
O17—H17⋯O11	0.82	2.14	2.926 (4)	160
O16—H16⋯O17	0.85	1.89	2.730 (5)	170
O15—H25⋯O16^i^	0.85	1.83	2.660 (4)	169
O14—H24⋯O6^ii^	0.84	2.25	3.090 (4)	173

Symmetry codes: (i) 
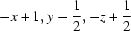
; (ii) 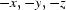.

**Fig. 1.**
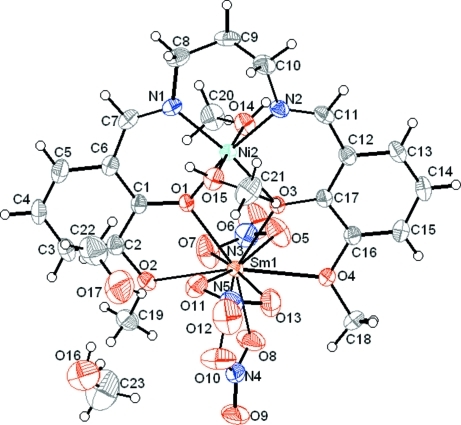
The molecular structure of (I), showing 40% probability displacement ellipsoids.

## supplementary crystallographic information

### Comment

As shown in Fig. 1, the hexadentate Schiff base ligand links Ni and Sm atoms into a dinuclear complex through two phenolate O atoms, which is similar to the bonding reported for other Nickel-Praseodymium and copper-samarium complexes of the same ligand (Liu & Zhang, 2008 and Wang et al., 2008). The Sm^III^ ion in the title complex is ten-coordinated by four oxygen atoms from the ligand and six oxygen atoms from three nitrate ions. The Ni^II^ center is six-coordinate by two nitrogen atoms and two oxygen atoms from the ligand and two methanol oxygen atoms. There are two solvent methanol molecules in the asymmetric unit. In the crystal structure, intermolecular O—H—O hydrogen bonds connect complex molecules and methanol solvent molecules to form (10-2) sheets.

### Experimental

The title complex was obtained by the treatment of nickel(II) acetate tetrahydrate (0.0622 g, 0.25 mmol) with the Schiff base (0.0855 g, 0.25 mmol) in methanol (25 ml) at room temperature. The mixture was refluxed for 3 h after the addition of samarium(III) nitrate hexahydrate (0.1115 g, 0.25 mmol). The reaction mixture was cooled and filtered; diethyl ether was allowed to diffuse slowly into the solution of the filtrate. Blue single crystals were obtained after several days. Analysis calculated for C_23_H_36_NiN_5_O_17_ Sm: C31.99; H, 4.20; N, 8.11; found: C, 32.01; H, 4.18; N, 8.14

### Refinement

H atoms bound to C atoms were placed in calculated positions and treated as riding on their parent atoms, with C—H = 0.93 Å (aromatic C), C—H = 0.97 Å (methylene C), C—H = 0.98 Å (methine C), and with *U*_iso_(H) = 1.2U_eq_(C) or C—H = 0.96 Å (methly C) and with *U*_iso_(H) = 1.5U_eq_(C). H atoms bonded to O atoms were placed in calculated positions and treated as riding on their parent atoms, with O—H = 0.82-0.85 Å, and with *U*_iso_(H) = 1.5U_eq_(O).

### Crystal data

**Table:**

[NiSm(C_19_H_20_N_2_O_4_)(NO_3_)_3_(CH_4_O)_2_]·2CH_4_O	*F*(000) = 1740
*M**_r_* = 863.63	*D*_x_ = 1.784 Mg m^−^^3^
Monoclinic, *P*2_1_/*c*	Mo *K*α radiation, λ = 0.71073 Å
Hall symbol: -P 2ybc	Cell parameters from 19157 reflections
*a* = 13.066 (3) Å	θ = 3.0–27.5°
*b* = 11.121 (2) Å	µ = 2.48 mm^−^^1^
*c* = 22.128 (4) Å	*T* = 291 K
β = 90.60 (3)°	Block, green
*V* = 3215.2 (11) Å^3^	0.21 × 0.20 × 0.18 mm
*Z* = 4	

### Data collection

**Table:**

Rigaku R-AXIS RAPID diffractometer	7354 independent reflections
Radiation source: fine-focus sealed tube	6200 reflections with *I* > 2σ(*I*)
graphite	*R*_int_ = 0.035
ω scans	θ_max_ = 27.5°, θ_min_ = 3.0°
Absorption correction: multi-scan (*ABSCOR*; Higashi, 1995)	*h* = −16→16
*T*_min_ = 0.619, *T*_max_ = 0.660	*k* = −12→14
22609 measured reflections	*l* = −24→28

### Refinement

**Table:**

Refinement on *F*^2^	Primary atom site location: structure-invariant direct methods
Least-squares matrix: full	Secondary atom site location: difference Fourier map
*R*[*F*^2^ > 2σ(*F*^2^)] = 0.027	Hydrogen site location: inferred from neighbouring sites
*wR*(*F*^2^) = 0.063	H-atom parameters constrained
*S* = 1.03	*w* = 1/[σ^2^(*F*_o_^2^) + (0.0241*P*)^2^ + 2.0539*P*] where *P* = (*F*_o_^2^ + 2*F*_c_^2^)/3
7354 reflections	(Δ/σ)_max_ = 0.017
430 parameters	Δρ_max_ = 0.58 e Å^−^^3^
18 restraints	Δρ_min_ = −0.38 e Å^−^^3^

### Special details

**Table:**

Geometry. All e.s.d.'s (except the e.s.d. in the dihedral angle between two l.s. planes) are estimated using the full covariance matrix. The cell e.s.d.'s are taken into account individually in the estimation of e.s.d.'s in distances, angles and torsion angles; correlations between e.s.d.'s in cell parameters are only used when they are defined by crystal symmetry. An approximate (isotropic) treatment of cell e.s.d.'s is used for estimating e.s.d.'s involving l.s. planes.
Refinement. Refinement of *F*^2^ against ALL reflections. The weighted *R*-factor *wR* and goodness of fit *S* are based on *F*^2^, conventional *R*-factors *R* are based on *F*, with *F* set to zero for negative *F*^2^. The threshold expression of *F*^2^ > σ(*F*^2^) is used only for calculating *R*-factors(gt) *etc*. and is not relevant to the choice of reflections for refinement. *R*-factors based on *F*^2^ are statistically about twice as large as those based on *F*, and *R*- factors based on ALL data will be even larger.

### Fractional atomic coordinates and isotropic or equivalent isotropic displacement parameters (Å^2^)

**Table:**

	*x*	*y*	*z*	*U*_iso_*/*U*_eq_	
C1	0.37696 (19)	0.0687 (2)	0.09085 (12)	0.0307 (6)	
C2	0.4451 (2)	0.1483 (3)	0.06351 (13)	0.0356 (6)	
C3	0.5471 (2)	0.1224 (3)	0.05614 (15)	0.0466 (8)	
H3	0.5902	0.1773	0.0376	0.056*	
C4	0.5847 (2)	0.0131 (3)	0.07685 (17)	0.0531 (9)	
H4	0.6537	−0.0052	0.0727	0.064*	
C5	0.5206 (2)	−0.0671 (3)	0.10321 (15)	0.0483 (8)	
H5	0.5468	−0.1401	0.1168	0.058*	
C6	0.4157 (2)	−0.0426 (3)	0.11042 (13)	0.0362 (6)	
C7	0.3549 (2)	−0.1364 (3)	0.13802 (13)	0.0394 (7)	
H7	0.3883	−0.2090	0.1450	0.047*	
C8	0.2241 (3)	−0.2432 (3)	0.18445 (17)	0.0530 (9)	
H8A	0.2521	−0.2457	0.2252	0.064*	
H8B	0.2499	−0.3127	0.1629	0.064*	
C9	0.1098 (3)	−0.2518 (3)	0.18747 (16)	0.0505 (8)	
H9A	0.0821	−0.2481	0.1467	0.061*	
H9B	0.0919	−0.3296	0.2041	0.061*	
C10	0.0596 (3)	−0.1561 (3)	0.22451 (16)	0.0495 (8)	
H10A	−0.0075	−0.1837	0.2368	0.059*	
H10B	0.1002	−0.1418	0.2608	0.059*	
C11	−0.0399 (2)	0.0033 (3)	0.18723 (13)	0.0373 (6)	
H11	−0.0922	−0.0389	0.2059	0.045*	
C12	−0.0688 (2)	0.1147 (3)	0.15761 (12)	0.0337 (6)	
C13	−0.1738 (2)	0.1418 (3)	0.15547 (14)	0.0419 (7)	
H13	−0.2202	0.0871	0.1713	0.050*	
C14	−0.2094 (2)	0.2461 (3)	0.13083 (15)	0.0466 (8)	
H14	−0.2794	0.2611	0.1289	0.056*	
C15	−0.1405 (2)	0.3301 (3)	0.10859 (14)	0.0412 (7)	
H15	−0.1640	0.4022	0.0923	0.049*	
C16	−0.0381 (2)	0.3060 (2)	0.11079 (12)	0.0320 (6)	
C17	0.00114 (19)	0.1967 (2)	0.13375 (12)	0.0289 (5)	
C18	0.0083 (3)	0.5082 (3)	0.0866 (2)	0.0605 (10)	
H18A	−0.0408	0.5189	0.0544	0.091*	
H18B	0.0678	0.5560	0.0787	0.091*	
H18C	−0.0213	0.5329	0.1241	0.091*	
C19	0.4551 (3)	0.3289 (3)	0.00308 (18)	0.0585 (10)	
H19A	0.5073	0.3734	0.0242	0.088*	
H19B	0.4087	0.3837	−0.0164	0.088*	
H19C	0.4862	0.2783	−0.0267	0.088*	
C20	0.1502 (3)	−0.0970 (4)	0.01411 (16)	0.0600 (10)	
H20A	0.2163	−0.0603	0.0104	0.090*	
H20B	0.1102	−0.0813	−0.0217	0.090*	
H20C	0.1582	−0.1823	0.0191	0.090*	
C21	0.1855 (3)	0.1427 (4)	0.27373 (18)	0.0701 (11)	
H21A	0.1321	0.1907	0.2559	0.105*	
H21B	0.2320	0.1937	0.2958	0.105*	
H21C	0.1561	0.0851	0.3008	0.105*	
C22	0.5326 (4)	0.1673 (5)	0.2244 (2)	0.0843 (14)	
H22A	0.5915	0.1358	0.2453	0.126*	
H22B	0.5473	0.1754	0.1822	0.126*	
H22C	0.4758	0.1135	0.2294	0.126*	
C23	0.5862 (5)	0.4994 (6)	0.1504 (3)	0.121 (2)	
H23A	0.5258	0.5453	0.1589	0.182*	
H23B	0.5684	0.4323	0.1252	0.182*	
H23C	0.6348	0.5493	0.1301	0.182*	
N1	0.26110 (18)	−0.1320 (2)	0.15402 (11)	0.0353 (5)	
N2	0.04886 (17)	−0.0434 (2)	0.19060 (11)	0.0346 (5)	
N3	0.1429 (2)	0.2064 (3)	−0.04335 (14)	0.0541 (7)	
N4	0.2728 (2)	0.5403 (3)	0.03306 (15)	0.0537 (8)	
N5	0.2758 (2)	0.3905 (3)	0.19970 (14)	0.0529 (7)	
Ni2	0.17216 (2)	0.01724 (3)	0.146022 (15)	0.02827 (8)	
O1	0.27952 (13)	0.10168 (16)	0.09603 (8)	0.0311 (4)	
O2	0.40017 (15)	0.25598 (19)	0.04516 (10)	0.0412 (5)	
O3	0.10161 (13)	0.17856 (16)	0.13255 (9)	0.0311 (4)	
O4	0.03661 (14)	0.38477 (17)	0.09025 (9)	0.0385 (5)	
O5	0.08672 (19)	0.2286 (3)	−0.00061 (12)	0.0668 (7)	
O6	0.1076 (3)	0.1702 (4)	−0.09119 (14)	0.0976 (11)	
O7	0.2361 (2)	0.2184 (3)	−0.03317 (14)	0.0818 (9)	
O8	0.2134 (2)	0.4692 (2)	0.00560 (14)	0.0749 (9)	
O9	0.2952 (2)	0.6379 (3)	0.01348 (17)	0.0914 (11)	
O10	0.3109 (3)	0.5005 (3)	0.07983 (14)	0.0843 (9)	
O11	0.33076 (18)	0.3210 (2)	0.16908 (11)	0.0501 (6)	
O12	0.3047 (2)	0.4284 (3)	0.24826 (14)	0.0928 (11)	
O13	0.19013 (18)	0.4171 (2)	0.17730 (13)	0.0656 (7)	
O14	0.09997 (16)	−0.0488 (2)	0.06488 (9)	0.0441 (5)	
H24	0.0436	−0.0842	0.0689	0.066*	
O15	0.23897 (16)	0.0819 (2)	0.22773 (9)	0.0447 (5)	
H25	0.2843	0.0390	0.2448	0.067*	
O16	0.6290 (3)	0.4577 (3)	0.20449 (14)	0.0923 (10)	
H16	0.5979	0.3977	0.2191	0.138*	
O17	0.5084 (3)	0.2774 (3)	0.24771 (19)	0.1081 (12)	
H17	0.4614	0.3070	0.2279	0.162*	
Sm1	0.216551 (10)	0.295997 (12)	0.078087 (6)	0.02961 (5)	

### Atomic displacement parameters (Å^2^)

**Table:**

	*U*^11^	*U*^22^	*U*^33^	*U*^12^	*U*^13^	*U*^23^
C1	0.0272 (12)	0.0361 (14)	0.0289 (14)	0.0032 (11)	−0.0008 (10)	−0.0050 (11)
C2	0.0315 (14)	0.0414 (16)	0.0340 (15)	0.0013 (12)	0.0020 (11)	−0.0055 (12)
C3	0.0281 (14)	0.060 (2)	0.052 (2)	−0.0038 (14)	0.0049 (13)	−0.0033 (16)
C4	0.0284 (15)	0.066 (2)	0.065 (2)	0.0122 (15)	0.0047 (14)	−0.0039 (18)
C5	0.0380 (16)	0.0513 (19)	0.056 (2)	0.0143 (15)	−0.0020 (14)	−0.0043 (16)
C6	0.0349 (14)	0.0387 (15)	0.0350 (15)	0.0063 (12)	−0.0006 (12)	−0.0036 (12)
C7	0.0478 (17)	0.0326 (15)	0.0378 (16)	0.0100 (13)	−0.0052 (13)	−0.0013 (12)
C8	0.061 (2)	0.0345 (16)	0.063 (2)	0.0054 (16)	0.0026 (17)	0.0153 (16)
C9	0.066 (2)	0.0296 (14)	0.056 (2)	−0.0134 (16)	−0.0071 (17)	0.0117 (15)
C10	0.0487 (18)	0.0475 (18)	0.052 (2)	−0.0064 (15)	0.0076 (15)	0.0205 (16)
C11	0.0372 (15)	0.0406 (15)	0.0342 (16)	−0.0120 (13)	0.0084 (12)	0.0006 (12)
C12	0.0313 (13)	0.0396 (15)	0.0304 (14)	−0.0026 (12)	0.0035 (11)	−0.0020 (12)
C13	0.0279 (14)	0.0530 (19)	0.0450 (18)	−0.0065 (13)	0.0058 (12)	−0.0022 (14)
C14	0.0253 (14)	0.063 (2)	0.051 (2)	0.0048 (14)	0.0001 (13)	−0.0014 (16)
C15	0.0335 (14)	0.0459 (17)	0.0443 (18)	0.0093 (13)	0.0002 (12)	−0.0002 (14)
C16	0.0300 (13)	0.0355 (14)	0.0305 (14)	0.0001 (11)	0.0000 (10)	−0.0018 (11)
C17	0.0274 (12)	0.0330 (14)	0.0264 (13)	0.0004 (11)	0.0002 (10)	−0.0051 (11)
C18	0.051 (2)	0.0308 (16)	0.100 (3)	0.0085 (15)	0.0126 (19)	0.0023 (18)
C19	0.053 (2)	0.0515 (19)	0.071 (3)	−0.0058 (17)	0.0269 (18)	0.0134 (18)
C20	0.062 (2)	0.073 (2)	0.045 (2)	−0.0017 (19)	0.0036 (16)	−0.0195 (18)
C21	0.088 (3)	0.068 (3)	0.054 (2)	0.019 (2)	0.002 (2)	−0.018 (2)
C22	0.075 (3)	0.094 (4)	0.083 (3)	−0.013 (3)	−0.008 (2)	−0.021 (3)
C23	0.122 (5)	0.154 (6)	0.087 (4)	0.029 (4)	−0.046 (4)	−0.009 (4)
N1	0.0423 (13)	0.0290 (12)	0.0344 (13)	0.0023 (10)	−0.0017 (10)	0.0024 (10)
N2	0.0346 (12)	0.0348 (12)	0.0344 (13)	−0.0045 (10)	0.0032 (10)	0.0071 (10)
N3	0.0525 (17)	0.0591 (18)	0.0505 (18)	−0.0029 (14)	−0.0134 (14)	−0.0111 (14)
N4	0.0414 (15)	0.0422 (15)	0.078 (2)	0.0068 (13)	0.0100 (14)	0.0231 (15)
N5	0.0504 (17)	0.0560 (18)	0.0524 (18)	−0.0085 (14)	0.0000 (13)	−0.0128 (14)
Ni2	0.02844 (16)	0.02665 (17)	0.02974 (18)	−0.00105 (13)	0.00119 (13)	0.00296 (14)
O1	0.0233 (8)	0.0332 (10)	0.0368 (10)	0.0009 (8)	0.0033 (7)	0.0032 (8)
O2	0.0325 (10)	0.0402 (11)	0.0511 (13)	−0.0038 (9)	0.0105 (9)	0.0085 (10)
O3	0.0231 (8)	0.0296 (9)	0.0407 (11)	0.0016 (7)	0.0036 (7)	0.0041 (8)
O4	0.0308 (10)	0.0309 (10)	0.0539 (13)	0.0045 (8)	0.0021 (9)	0.0067 (9)
O5	0.0522 (14)	0.099 (2)	0.0495 (16)	−0.0161 (14)	0.0033 (12)	−0.0099 (14)
O6	0.085 (2)	0.146 (3)	0.0609 (19)	−0.013 (2)	−0.0192 (16)	−0.044 (2)
O7	0.0520 (15)	0.117 (3)	0.076 (2)	−0.0024 (16)	−0.0034 (14)	−0.0299 (18)
O8	0.0649 (17)	0.0625 (17)	0.097 (2)	−0.0198 (14)	−0.0206 (15)	0.0406 (16)
O9	0.0687 (18)	0.0565 (17)	0.149 (3)	−0.0134 (15)	0.0006 (18)	0.0539 (19)
O10	0.124 (3)	0.0562 (16)	0.072 (2)	−0.0263 (17)	−0.0174 (18)	0.0133 (15)
O11	0.0521 (13)	0.0475 (13)	0.0505 (14)	0.0078 (11)	−0.0107 (11)	−0.0072 (11)
O12	0.083 (2)	0.127 (3)	0.068 (2)	−0.005 (2)	−0.0138 (16)	−0.052 (2)
O13	0.0444 (13)	0.0747 (17)	0.0777 (18)	0.0055 (13)	−0.0018 (12)	−0.0232 (15)
O14	0.0404 (11)	0.0554 (13)	0.0366 (12)	−0.0053 (10)	0.0002 (9)	−0.0073 (10)
O15	0.0466 (12)	0.0498 (13)	0.0374 (12)	0.0042 (10)	−0.0059 (9)	−0.0053 (10)
O16	0.100 (2)	0.092 (2)	0.084 (2)	−0.0052 (19)	−0.0437 (19)	−0.0032 (18)
O17	0.079 (2)	0.109 (3)	0.135 (3)	0.009 (2)	−0.041 (2)	−0.008 (2)
Sm1	0.02562 (7)	0.02790 (8)	0.03532 (8)	−0.00162 (6)	0.00024 (5)	0.00431 (6)

### Geometric parameters (Å, °)

**Table:**

C1—O1	1.331 (3)	C20—O14	1.413 (4)
C1—C2	1.397 (4)	C20—H20A	0.9600
C1—C6	1.404 (4)	C20—H20B	0.9600
C2—C3	1.375 (4)	C20—H20C	0.9600
C2—O2	1.393 (4)	C21—O15	1.413 (4)
C3—C4	1.387 (5)	C21—H21A	0.9600
C3—H3	0.9300	C21—H21B	0.9600
C4—C5	1.359 (5)	C21—H21C	0.9600
C4—H4	0.9300	C22—O17	1.367 (6)
C5—C6	1.409 (4)	C22—H22A	0.9600
C5—H5	0.9300	C22—H22B	0.9600
C6—C7	1.450 (4)	C22—H22C	0.9600
C7—N1	1.280 (4)	C23—O16	1.394 (6)
C7—H7	0.9300	C23—H23A	0.9600
C8—N1	1.490 (4)	C23—H23B	0.9600
C8—C9	1.499 (5)	C23—H23C	0.9600
C8—H8A	0.9700	N1—Ni2	2.033 (2)
C8—H8B	0.9700	N2—Ni2	2.014 (2)
C9—C10	1.498 (5)	N3—O6	1.219 (4)
C9—H9A	0.9700	N3—O5	1.228 (4)
C9—H9B	0.9700	N3—O7	1.242 (4)
C10—N2	1.467 (4)	N4—O9	1.206 (3)
C10—H10A	0.9700	N4—O10	1.226 (4)
C10—H10B	0.9700	N4—O8	1.261 (4)
C11—N2	1.272 (4)	N5—O12	1.211 (4)
C11—C12	1.450 (4)	N5—O13	1.254 (4)
C11—H11	0.9300	N5—O11	1.258 (3)
C12—C17	1.399 (4)	Ni2—O1	2.0262 (18)
C12—C13	1.406 (4)	Ni2—O3	2.0375 (18)
C13—C14	1.361 (5)	Ni2—O15	2.125 (2)
C13—H13	0.9300	Ni2—O14	2.149 (2)
C14—C15	1.391 (5)	O1—Sm1	2.3448 (18)
C14—H14	0.9300	O2—Sm1	2.554 (2)
C15—C16	1.365 (4)	O3—Sm1	2.3349 (18)
C15—H15	0.9300	O4—Sm1	2.5668 (19)
C16—O4	1.392 (3)	O5—Sm1	2.532 (3)
C16—C17	1.411 (4)	O7—Sm1	2.624 (3)
C17—O3	1.329 (3)	O8—Sm1	2.506 (2)
C18—O4	1.424 (4)	O10—Sm1	2.587 (3)
C18—H18A	0.9600	O11—Sm1	2.509 (2)
C18—H18B	0.9600	O13—Sm1	2.602 (3)
C18—H18C	0.9600	O14—H24	0.8409
C19—O2	1.433 (4)	O15—H25	0.8461
C19—H19A	0.9600	O16—H16	0.8476
C19—H19B	0.9600	O17—H17	0.8200
C19—H19C	0.9600		
			
O1—C1—C2	118.5 (2)	C11—N2—C10	117.5 (2)
O1—C1—C6	123.9 (2)	C11—N2—Ni2	124.6 (2)
C2—C1—C6	117.6 (2)	C10—N2—Ni2	117.63 (19)
C3—C2—O2	123.5 (3)	O6—N3—O5	120.7 (3)
C3—C2—C1	122.8 (3)	O6—N3—O7	123.6 (3)
O2—C2—C1	113.7 (2)	O5—N3—O7	115.6 (3)
C2—C3—C4	119.0 (3)	O9—N4—O10	122.1 (4)
C2—C3—H3	120.5	O9—N4—O8	122.8 (3)
C4—C3—H3	120.5	O10—N4—O8	115.0 (3)
C5—C4—C3	120.0 (3)	O12—N5—O13	122.5 (3)
C5—C4—H4	120.0	O12—N5—O11	121.1 (3)
C3—C4—H4	120.0	O13—N5—O11	116.4 (3)
C4—C5—C6	121.7 (3)	N2—Ni2—O1	169.87 (8)
C4—C5—H5	119.1	N2—Ni2—N1	98.25 (10)
C6—C5—H5	119.1	O1—Ni2—N1	91.58 (9)
C1—C6—C5	118.9 (3)	N2—Ni2—O3	90.19 (8)
C1—C6—C7	124.5 (3)	O1—Ni2—O3	80.03 (7)
C5—C6—C7	116.6 (3)	N1—Ni2—O3	171.50 (9)
N1—C7—C6	128.3 (3)	N2—Ni2—O15	91.15 (9)
N1—C7—H7	115.9	O1—Ni2—O15	91.56 (8)
C6—C7—H7	115.9	N1—Ni2—O15	88.44 (9)
N1—C8—C9	113.6 (3)	O3—Ni2—O15	90.45 (8)
N1—C8—H8A	108.8	N2—Ni2—O14	87.04 (9)
C9—C8—H8A	108.8	O1—Ni2—O14	90.15 (8)
N1—C8—H8B	108.8	N1—Ni2—O14	92.23 (9)
C9—C8—H8B	108.8	O3—Ni2—O14	89.14 (8)
H8A—C8—H8B	107.7	O15—Ni2—O14	178.14 (8)
C10—C9—C8	114.9 (3)	C1—O1—Ni2	126.00 (17)
C10—C9—H9A	108.5	C1—O1—Sm1	124.99 (16)
C8—C9—H9A	108.5	Ni2—O1—Sm1	106.01 (7)
C10—C9—H9B	108.5	C2—O2—C19	117.6 (2)
C8—C9—H9B	108.5	C2—O2—Sm1	117.45 (15)
H9A—C9—H9B	107.5	C19—O2—Sm1	124.43 (19)
N2—C10—C9	111.5 (3)	C17—O3—Ni2	125.21 (16)
N2—C10—H10A	109.3	C17—O3—Sm1	124.48 (16)
C9—C10—H10A	109.3	Ni2—O3—Sm1	106.00 (7)
N2—C10—H10B	109.3	C16—O4—C18	116.3 (2)
C9—C10—H10B	109.3	C16—O4—Sm1	116.01 (15)
H10A—C10—H10B	108.0	C18—O4—Sm1	127.06 (18)
N2—C11—C12	127.5 (3)	N3—O5—Sm1	100.79 (19)
N2—C11—H11	116.3	N3—O7—Sm1	95.8 (2)
C12—C11—H11	116.3	N4—O8—Sm1	99.7 (2)
C17—C12—C13	119.3 (3)	N4—O10—Sm1	96.7 (2)
C17—C12—C11	124.1 (2)	N5—O11—Sm1	99.32 (18)
C13—C12—C11	116.6 (3)	N5—O13—Sm1	94.90 (19)
C14—C13—C12	121.7 (3)	C20—O14—Ni2	126.2 (2)
C14—C13—H13	119.2	C20—O14—H24	108.8
C12—C13—H13	119.2	Ni2—O14—H24	116.6
C13—C14—C15	119.6 (3)	C21—O15—Ni2	125.0 (2)
C13—C14—H14	120.2	C21—O15—H25	107.2
C15—C14—H14	120.2	Ni2—O15—H25	118.0
C16—C15—C14	119.6 (3)	C23—O16—H16	113.6
C16—C15—H15	120.2	C22—O17—H17	109.4
C14—C15—H15	120.2	O3—Sm1—O1	67.88 (6)
C15—C16—O4	123.7 (3)	O3—Sm1—O8	138.92 (8)
C15—C16—C17	122.3 (3)	O1—Sm1—O8	145.08 (9)
O4—C16—C17	114.0 (2)	O3—Sm1—O11	91.61 (8)
O3—C17—C12	123.9 (2)	O1—Sm1—O11	76.20 (7)
O3—C17—C16	118.6 (2)	O8—Sm1—O11	115.73 (9)
C12—C17—C16	117.5 (2)	O3—Sm1—O5	76.08 (8)
O4—C18—H18A	109.5	O1—Sm1—O5	94.29 (8)
O4—C18—H18B	109.5	O8—Sm1—O5	77.35 (10)
H18A—C18—H18B	109.5	O11—Sm1—O5	166.73 (9)
O4—C18—H18C	109.5	O3—Sm1—O2	131.38 (6)
H18A—C18—H18C	109.5	O1—Sm1—O2	63.81 (6)
H18B—C18—H18C	109.5	O8—Sm1—O2	87.74 (8)
O2—C19—H19A	109.5	O11—Sm1—O2	72.28 (8)
O2—C19—H19B	109.5	O5—Sm1—O2	112.14 (8)
H19A—C19—H19B	109.5	O3—Sm1—O4	64.29 (6)
O2—C19—H19C	109.5	O1—Sm1—O4	131.06 (6)
H19A—C19—H19C	109.5	O8—Sm1—O4	76.27 (8)
H19B—C19—H19C	109.5	O11—Sm1—O4	114.27 (7)
O14—C20—H20A	109.5	O5—Sm1—O4	65.05 (8)
O14—C20—H20B	109.5	O2—Sm1—O4	164.00 (6)
H20A—C20—H20B	109.5	O3—Sm1—O10	142.47 (9)
O14—C20—H20C	109.5	O1—Sm1—O10	129.90 (9)
H20A—C20—H20C	109.5	O8—Sm1—O10	48.60 (9)
H20B—C20—H20C	109.5	O11—Sm1—O10	67.13 (9)
O15—C21—H21A	109.5	O5—Sm1—O10	125.90 (10)
O15—C21—H21B	109.5	O2—Sm1—O10	73.05 (9)
H21A—C21—H21B	109.5	O4—Sm1—O10	95.58 (9)
O15—C21—H21C	109.5	O3—Sm1—O13	76.25 (8)
H21A—C21—H21C	109.5	O1—Sm1—O13	112.56 (8)
H21B—C21—H21C	109.5	O8—Sm1—O13	98.09 (10)
O17—C22—H22A	109.5	O11—Sm1—O13	49.35 (8)
O17—C22—H22B	109.5	O5—Sm1—O13	129.78 (8)
H22A—C22—H22B	109.5	O2—Sm1—O13	117.67 (8)
O17—C22—H22C	109.5	O4—Sm1—O13	65.33 (8)
H22A—C22—H22C	109.5	O10—Sm1—O13	66.40 (10)
H22B—C22—H22C	109.5	O3—Sm1—O7	111.70 (9)
O16—C23—H23A	109.5	O1—Sm1—O7	79.53 (9)
O16—C23—H23B	109.5	O8—Sm1—O7	69.72 (11)
H23A—C23—H23B	109.5	O11—Sm1—O7	136.60 (8)
O16—C23—H23C	109.5	O5—Sm1—O7	47.80 (9)
H23A—C23—H23C	109.5	O2—Sm1—O7	64.79 (8)
H23B—C23—H23C	109.5	O4—Sm1—O7	108.85 (8)
C7—N1—C8	114.2 (2)	O10—Sm1—O7	104.61 (10)
C7—N1—Ni2	123.7 (2)	O13—Sm1—O7	167.72 (10)
C8—N1—Ni2	121.9 (2)		

### Hydrogen-bond geometry (Å, °)

**Table:**

*D*—H···*A*	*D*—H	H···*A*	*D*···*A*	*D*—H···*A*
O17—H17···O11	0.82	2.14	2.926 (4)	160
O16—H16···O17	0.85	1.89	2.730 (5)	170
O15—H25···O16^i^	0.85	1.83	2.660 (4)	169
O14—H24···O6^ii^	0.84	2.25	3.090 (4)	173

Symmetry codes: (i) −*x*+1, *y*−1/2, −*z*+1/2; (ii) −*x*, −*y*, −*z*.
